# Human Papillomavirus Vaccination Uptake in the Rio Grande Valley: Results from a Pilot Community-Based Educational and School-Based Vaccination Program and Its Expansion

**DOI:** 10.3390/vaccines11020329

**Published:** 2023-02-01

**Authors:** Ana M. Rodriguez, Thuy Quynh N. Do, Mostafa F. Eyada, Lu Chen, Kathleen M. Schmeler, Jane R. Montealegre

**Affiliations:** 1 Department of Obstetrics and Gynecology, University of Texas Medical Branch, Galveston, TX 77555, USA; 2 Department of Preventive Medicine and Population Health, University of Texas Medical Branch, Galveston, TX 77555, USA; 3 Office of Biostatistics, Preventive Medicine and Population Health, University of Texas Medical Branch, Galveston, TX 77555, USA; 4 Department of Gynecologic Oncology and Reproductive Medicine, The University of Texas MD Anderson Cancer Center, Houston, TX 77030, USA; 5 School of Health Professions, Dan L Duncan Comprehensive Cancer Center, Baylor College of Medicine, Houston, TX 77030, USA

**Keywords:** human papillomavirus vaccine, adolescent health, human papillomavirus-related cancers and diseases, school-based vaccination, provider recommendation, Rio Grande Valley

## Abstract

Human papillomavirus (HPV) vaccine is a safe and effective strategy for reducing HPV morbidity and mortality. Schools have become an increasingly attractive setting for delivering vaccinations and supporting vaccination health literacy and decisional support. This study assesses the effectiveness of a community-based, physician-led HPV education campaign (starting in 2016) and onsite middle school-based HPV vaccination program across six school districts (2017, 2019, 2020) in a rural, medically underserved Texas area (Rio Grande Valley). Pre- and post-intervention HPV vaccination rates were tracked against the 2016 National Immunization Survey—Teen target rates (initiation: 49.3%; completion: 32.9%). Summary statistics were stratified by gender, school district, and grade level. The study reached 19,951 students who received HPV vaccines directly or indirectly through our program (10,289 females; 9662 males) (August 2016–August 2022). Of those, 2145 students (1074 females; 1071 males) were vaccinated directly through our program. The overall HPV up-to-date (UTD) rates were 58.8%. The overall median age at HPV vaccine initiation and HPV-UTD (range) was 11 years (9–21) and 12 years (9–20). The overall median interval between HPV vaccine doses (range) was 291 days (146–2968). Recommending HPV vaccine initiation at younger ages increases HPV vaccine completion and providing access to HPV vaccines encourages on-time vaccination and completion.

## 1. Introduction

Human papillomavirus (HPV) vaccinations have proven to be a valuable, cost-effective public health intervention for reducing HPV morbidity and mortality [1]. However, HPV vaccine uptake among adolescents in the United States (US) is far below the Healthy People 2020 goal of 80% (51.1% completion rate). Routine HPV vaccination in the US has been recommended for females since 2006 and for males aged 9–26 years since 2011. Although HPV vaccination is recommended for adolescents aged 11–12, it can be initiated as early as 9 years of age [2]. According to the 2016 Advisory Committee on Immunization Practices (ACIP) guidelines, HPV-UTD is defined as either: (1) receipt of 3 or more doses or (2) receipt of 2 doses of the HPV vaccine, with the first shot administered before age 15 years, and the time between the first and second dose being at least 5 months minus 4 days.

Personal, community, and provider-level factors may inhibit the translation of the willingness to vaccinate into an actual HPV vaccine update. Acceptance may not be the only precursor of the willingness to vaccinate. The most commonly cited reasons for low HPV vaccination rates in the US are missed clinical opportunities and the lack of strong and consistent vaccine recommendations from healthcare providers [3]. Other known factors affecting US HPV vaccine uptake include social norms of behavior, knowledge, provider recommendations and risk perception, accessibility, work /school schedule, insurance, and costs [4,5,6,7,8,9,10,11].

According to the National Immunization Survey—Teen (ages 13–17), Texas ranks as one of the lowest states in terms of HPV-UTD vaccination rates (47 out of 50 states and the District of Columbia) [12]. In 2016, the national average for HPV initiation and UTD was 60.4% and 43.4%, while Texas was 49.3% and 32.9%, respectively [13]. HPV vaccination rates are even lower in the rural areas of Texas. HPV-related diseases and cancers disproportionately affect low-income, rural, and minority individuals. In the Rio Grande Valley (RGV) of Texas, a rural, medically underserved area (four counties bordering Mexico: Cameron, Hidalgo, Starr, and Willacy Counties) [11,14,15], women have a 30% higher cervical cancer incidence and mortality rate compared with the rest of Texas [16,17]. Starr and Hidalgo Counties have especially high cervical cancer incidence and mortality rates [18] and a high proportion of uninsured residents [16,18,19]. Since rural communities often have a higher incidence and mortality of HPV-associated cancers and lower HPV vaccination rates [4], offering the HPV vaccine at no cost is important in the RGV. Residents are more likely to be Hispanic, medically underserved, less educated, have low health literacy, and be economically disadvantaged [20].

School-based vaccination programs are becoming a more widely considered method of delivering HPV immunizations to adolescent populations, with a grade-based vaccination strategy preferred over an age-based vaccination strategy [21,22]. Introduction of the HPV vaccine in a school-based setting provides a rare opportunity to build and strengthen adolescent health. Schools have become an increasingly attractive setting for delivering vaccinations because of their ability to reach a large number of children in a short period of time and reduce operational problems for parents [23,24]. School-based vaccination helps support vaccination health literacy and decisional involvement, reduces fear and anxiety, and increases access to needed health services, especially among medically underserved children and adolescents who may have limited encounters with healthcare providers [25,26,27]. This study assesses the effectiveness of a pilot community-based, physician-led HPV education campaign and an onsite, middle school-based HPV vaccination program, and the expansion to five additional school districts in a rural, medically underserved Texas area.

## 2. Materials and Methods

### 2.1. Study Setting, Design, and Period

This cross-sectional study summarizes multiple projects funded by the Cancer Prevention Research Institute of Texas (CPRIT) to increase HPV vaccine uptake in the RGV (Texas). The goals were to meet the 2016 NIS-Teen HPV vaccination rates (initiation: 49.3%; completion: 32.9%) [15,28,29]. The study period was from August 2016 to August 2022. The pilot project took place in Rio Grande City Consolidated Independent School District [RGCCISD] (3 middle schools) from 2016–2019 [11,15], and expanded into Pharr-San Juan-Alamo Independent School District [PSJA ISD] (8 middle schools) in 2019–2022 [29], and Roma ISD (2 middle schools), Zapata ISD (1 middle school), San Isidro ISD (1 middle school), and Jim Hogg ISD (1 middle school) in 2020–2022. Approval for this program was obtained from the University of Texas Medical Branch’s Institutional Review Board (IRB-19–0138 on August 21, 2019; IRB-21–0044 approved on March 24, 2021) and the school boards for RGCCISD, PSJA ISD, Roma ISD, Zapata ISD, San Isidro ISD, and Jim Hogg ISD. Parental consent was obtained prior to vaccination and documented in our system.

The study outcomes included HPV vaccine initiation and HPV-UTD status. For study inclusion, students received at least one HPV vaccine dose either directly (school-based vaccination program) or indirectly (physician referral or scheduled through patient navigators) through our program and had parental consent. HPV vaccine initiation was defined as receipt of the first dose of the HPV vaccine series. HPV-UTD was defined as receipt of ≥3 doses if initiated after age 15 years or with immunocompromising conditions, or receipt of 2 doses if initiated before age 15 years, with a minimum interval of 5 months between the first and second dose [30,31].

Results from a previously published survey were used to develop and strengthen strategies for implementing the two-component intervention to increase HPV vaccine uptake [11]. As described previously, this study combined community-based, physician-led HPV education with school-based vaccinations [11,15,28,29]. We targeted female and male middle school students at the recommended ages (aged 11–12 years of age), bundled the HPV vaccine with recommended vaccines (e.g., flu, meningococcal, meningitis B, tetanus, diphtheria [TD], or tetanus, diphtheria, and pertussis [TDAP] and hepatitis A vaccines), and addressed previously identified factors affecting HPV vaccine uptake (e.g., social norms, knowledge, health provider recommendations, and risk perception, accessibility, schedule, costs, and bundling vaccines) [4,5,6,7,9,10,11,15,32,33,34].

The physician-led educational events started in August 2016 in Cameron, Hidalgo, and Starr Counties (located in a 15-mile radius encompassing the pilot program in RGCCISD). In all school districts, school-based vaccinations were implemented with the largest student enrollments for the largest impact. Between 2019 and 2022, the school-based vaccination events were implemented in PSJA ISD (starting with middle schools with the largest enrollment in closest proximity to RGCCISD: August 2019 for Phase 1 [3 middle schools]; August 2020 for Phase 2 [3 middle schools]; and February 2021 for Phase 3 [2 middle schools]) [29]. Between 2020 and 2022 [29], the school-based vaccinations were implemented in Roma ISD in Starr County (2 middle schools), Zapata ISD (1 middle school) in Zapata County, San Isidro ISD (1 middle school) in Starr County, and Jim Hogg ISD (1 middle school) in Jim Hogg County. We collaborated with community and public health organizations to actively promote the school-based HPV vaccination program through stakeholder/PTA/school board meetings, social media, and radio. Although the target population included RGCCISD, PSJA ISD, Roma ISD, and Zapata County ISD middle school students, any student who came to vaccination events with parental consent and met the age criteria received their HPV vaccinations.

During the school year, the HPV vaccine series was initiated and completed at back-to-school events, progress report nights, and preview events. To ensure on-time vaccination and adherence to the dosing schedule, catch-up vaccination was scheduled by our staff through nearby clinics and planned events for missed doses. Up to 5 reminder letters, texts, and phone calls for subsequent doses were sent to the parents/guardians of children who initiated HPV vaccination. Prior to coronavirus disease 2019 (COVID-19), school-based vaccination events were held in the nurses’ offices, conference rooms, nearby clinics at parents’ requests, and at community events. Adaptations to how vaccinations were implemented were made when the COVID-19 pandemic hit and caused school closures during the first year of the school-based vaccination program in the expanded school districts [28,29]. We held outside events with social distancing, limited in-person activities, increased online activities, and provided more frequent stakeholder engagement through teleconferences, navigational services, and mobile van vaccinations [28].

### 2.2. Data Collection and Analysis

Given the transient nature of the student population in this area, the baseline cohort was followed for simplification. HPV vaccination rates are based on the baseline study cohort for each school. The HPV vaccination data was refreshed each quarter using data collected from the vaccine vendor and school immunization records (individual paper records) and reconciled with Immtrac2 (Texas Immunization Registry) [15,28,29]. The Texas Immunization Registry is secure and confidential, and it safely consolidates and stores immunization records from multiple sources in one centralized system. Summary statistics were computed and stratified by age of initiation (9–10, 11–12, 13–14, 15–16, 17+), gender, and school district.

Baseline HPV vaccination rates and demographic information (i.e., age, sex, and grade level of students) from the schools’ data processing departments were collected for the study cohort during the study period. The vendor collected student vaccination data (vaccine, dose number) during the vaccine administration. We tracked HPV vaccine administrations that were given directly through our vaccination program (i.e., school campus interventions and vaccination events) and those given indirectly through collaborating healthcare practices (awareness through our educational program and scheduled/referred to nearby clinics).

SAS version 9.4 (SAS Institute Inc., Cary, NC, USA) was used in conducting all analyses. Tables, graphs, and charts were used to perform descriptive analysis and report the frequencies. Logistic regression models were used to examine characteristics associated with HPV vaccine completion (HPV-UTD) using both school-based and non-school-based vaccination delivery modalities. Statistical significance was set at α| = |0.05 (two-sided).

## 3. Results

### 3.1. Descriptive Summary

A total of 19,951 students received HPV vaccines directly or indirectly through our program (10,289 females and 9662 males). Table 1 provides a summary of HPV vaccine activities in the pilot program in RGCCISD and the project’s expansion to five additional school districts (16 middle schools). We have conducted 178 school campus interventions. Across the 6 school districts, a total of 1549 HPV vaccine initiations and 1042 HPV vaccine completions (HPV-UTD) were delivered at school campuses. A total of 18,172 HPV vaccine initiations and 17,075 HPV vaccine completions were delivered through collaborating healthcare practices.

Table 2 displays the demographic characteristics of the study population and HPV vaccination rates by gender. Between August 2016 and August 2022, the study reached 19,951 students who received HPV vaccines directly or indirectly through our program across 6 school districts in the RGV (10,289 females and 9662 males). The overall HPV-UTD rate was 58.8%. Overall, the median age at HPV initiation and HPV-UTD (range) was 11 years (9–21) and 12 years (9–20). The median days between HPV vaccine doses (range) was 291 days (146–2968). The interval between HPV vaccine doses (range) was the shortest among the RGCCISD (Supplemental Table 1) at 268 days (14–2341).

Of the 19,951 students reached by our program, 2145 students (1074 females and 1071 males) were vaccinated directly (received at least 1 HPV vaccine dose) through our school-based vaccination program (Table 1 and Table A1). Most were from RGCCISD (45.1%; 968/2145) and PSJA ISD (44.6%; 957/2145). Most middle school students initiated the HPV vaccine at age 11–12 (69.5%, 1491/2145). The median age at HPV initiation and HPV-UTD (range) was 12 years (9–20) and 12 years (9–19). The median days between HPV vaccine doses (range) was 324 days (146–2855). Among the 2145 middle school students who received the HPV vaccine directly through our school-based program, 70.8% (1518/2145) had received the HPV vaccine bundled with other recommended vaccinations (Table 1). Among those who were directly vaccinated by our school-based program, the percentage of students who received their HPV vaccine with other recommended vaccinations was similar across female and male students (71.4% vs. 71.1%).

Table 3 shows the multivariate analysis of HPV-UTD for the overall cohort as well as for students directly vaccinated through our school-based program. In the overall cohort, a 1-year increase in age at initiation, being female, attending middle school at initiation, attending PSJA ISD, San Isidro ISD, and Zapata ISD, and receiving an initial dose through our program were significantly associated with HPV-UTD (*p*-value < 0.05). In this study, older age at initiation (1-year increase) was 32% less likely to be HPV-UTD (OR: 0.676, 95% confidence interval [95% CI]: 0.641–0.712) (Table 3). Male students were 10% less likely to be HPV-UTD compared to female students (OR: 0.902, 95% CI: 0.833–0.976). Compared to middle school students, elementary and high school students were 38–39% more likely to be HPV-UTD (OR: 1.385, 95% CI: 1.219–1.573; OR: 1.396, 95% CI: 1.097–1.776). Among students vaccinated by our program (Table 3), older age at initiation (1-year increase) was 37% less likely to be HPV-UTD (OR: 0.626, 95% CI: 0.549–0.714). Male students were 21% less likely to be HPV-UTD (OR: 0.792, 95% CI: 0.642–0.976) compared to female students. Compared to middle school students, high school students were 49% less likely to be HPV-UTD (OR: 0.517, 95% CI: 0.285–0.937).

### 3.2. HPV Initiation and HPV UTD Rates

**Figure 1:**
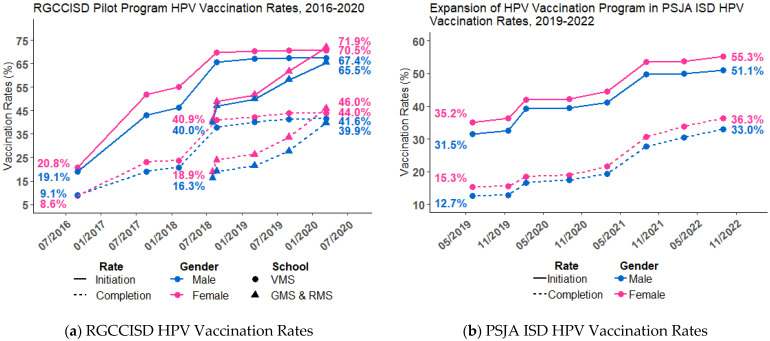
**HPV vaccination rates in the RGCCISD pilot program and its expansion in PSJA ISD**: (**a**) HPV vaccine initiation and HPV-UTD rates in the pilot program in RGCCISD from 2016–2020; (**b**) HPV vaccine initiation and HPV-UTD rates in the expansion in PSJA ISD.

Figure 1 shows the HPV vaccination rates in RGCCISD and PSJA ISD. Figure 1a shows the HPV vaccination rates by gender at baseline (2016, before the community-based education and school-based vaccination program) and at the end of the pilot program in RGCCISD. At baseline (2016), the HPV vaccine initiation and HPV-UTD rates for females at VMS (initial pilot middle school) were 20.8% and 8.6%. For males, the baseline HPV vaccine initiation and HPV-UTD rates at VMS were 19.1% and 9.1%. Between 2016 and 2020, the rates increased by almost four-fold to 70.5% and 44.0% among VMS female students and 67.4% and 41.6% among male students. For GMS and RMS, the baseline HPV vaccine initiation rate increased from 40.0% (male) and 40.9% (female) to 65.5% (male) and 71.9% (female). The HPV-UTD increased by 2.5-fold, from 16.3% (male) and 18.9% (female) to 41.6% (male) and 46.0% (female). Figure 1b shows the HPV vaccine initiation and HPV-UTD rates for the expansion of the HPV vaccination program in PSJA ISD (2019–2022) by gender. HPV initiation and HPV-UTD rates were slightly higher among female students compared to male students at baseline and at the end of the study period. HPV vaccine initiation rates increased from 35.2% to 55.3% for females and 15.3% to 36.3% for males. HPV-UTD (completion) increased from 15.3% to 36.3% for females and 12.7% to 33.3% for males.

**Figure 2:**
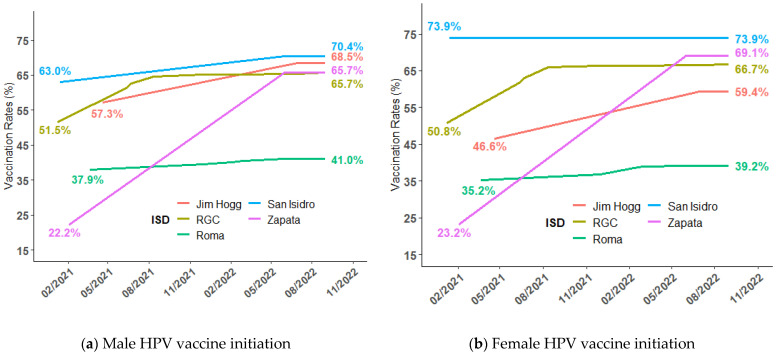

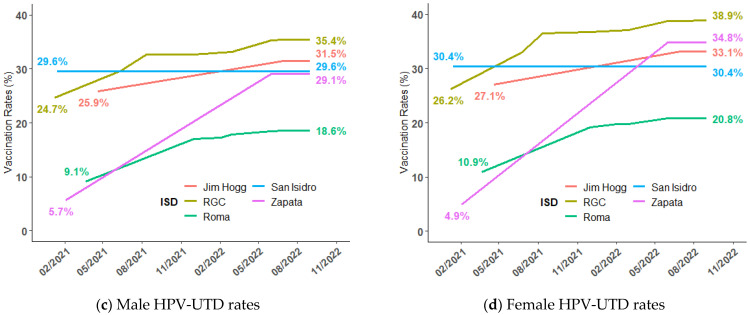
Male and female HPV vaccine initiations and HPV-UTD in RGCCISD, Roma ISD, San Isidro ISD, Zapata ISD, and Jim Hogg ISD: (**a**) Male HPV vaccine initiation in RGCCISD, Roma ISD, San Isidro ISD, Zapata ISD, and Jim Hogg ISD; (**b**) female HPV vaccine initiation in RGCCISD, Roma ISD, San Isidro ISD, Zapata ISD, and Jim Hogg ISD; (**c**) female HPV vaccine completion in RGCCISD, Roma ISD, San Isidro ISD, Zapata ISD, and Jim Hogg ISD; and (**d**) female HPV-UTD in RGCCISD, Roma ISD, San Isidro ISD, Zapata ISD, and Jim Hogg ISD.

Figure 2 shows the HPV vaccination rates in RGCCISD, Roma ISD, San Isidro ISD, Zapata ISD, and Jim Hogg ISD by gender. Baseline HPV vaccine initiation rates varied across school districts among males and females, with the highest in San Isidro ISD and the lowest in Zapata ISD. During the study period, Zapata ISD had the largest increase in male HPV vaccine initiation (increasing from 22.2% to 65.7%) and HPV-UTD rates (5.7% to 29.1%), while Roma ISD had the smallest increase in HPV vaccine initiation (37.9% to 41.0%). Among females, the largest increase in HPV vaccine initiation (23.2% to 69.1%) and HPV-UTD (4.9% to 34.8%) occurred in Zapata ISD. Regardless of gender, there was no change in HPV vaccine initiation or HPV-UTD rates among San Isidro ISD students.

## 4. Discussion

Over the last six years, our community-based education and school-based vaccination program has helped to build and strengthen adolescent health in the RGV [15,35]. The study results demonstrate how our community-based education and school-based vaccination program resulted in a high rate of HPV vaccine initiation and HPV-UTD. The goal was to meet the 2016 National Immunization Survey—Teen (NIS-Teen) HPV vaccination rates (49.3% for HPV vaccine initiation and 32.9% for HPV-UTD/completion [15,28,29]. We were able to surpass this goal across all six school districts. Between August 2016 and August 2022, 178 school-based interventions provided 2591 HPV vaccine doses and raised the overall HPV-UTD to 58.8% (exceeding the goal of 32.9%). The rate of HPV UTD was slightly higher among females compared to males (60.8% vs. 56.6%). A total of 1549 HPV vaccine initiations and 1042 HPV vaccine completions (HPV-UTD) were delivered to school campuses. A total of 18,172 HPV vaccine initiations and 17,075 HPV vaccine completions were delivered through collaborating healthcare practices. The overall median age at HPV vaccine initiation and HPV-UTD (range) was 11 years (9–21) and 12 (9–20). The overall median interval between HPV vaccine doses (range) was 291 days (146–2968). In the overall cohort, a 1-year increase in age at initiation, being female, attending middle school at initiation, attending PSJA ISD, San Isidro ISD, and Zapata ISD, and receiving an initial dose through our program were significantly associated with HPV-UTD (*p*-value < 0.05).

To our knowledge, our pilot project was one of the first school-based vaccination programs aimed at increasing HPV vaccination rates in RGV and Texas. Results from the pilot project in RGCCISD helped lay the foundation for the program by addressing known barriers affecting HPV vaccine uptake (e.g., social norms of behavior, knowledge, health provider recommendations and risk perception, accessibility, work /school schedule, costs, bundling HPV vaccines with other required vaccines) [4,5,6,7,8,9,10,11]. Besides removing known barriers, the results reinforce the importance of simplifying the messaging for HPV vaccination, recommending HPV vaccine initiation during early adolescence (age 11–12), and stressing the importance of on-time vaccination and adherence to the HPV vaccine schedule [36,37]. HPV vaccine uptake can be sustained if HPV vaccines are bundled with other required vaccines, and parents, local providers, school board members, and school staff are educated about its importance [38]. We were able to establish effective relationships and build trust between the school staff, school boards, local providers, health departments, and to some extent, the parents. Increased knowledge and positive perceptions of HPV vaccination are predictive of the vaccine’s acceptability [39] and reduce vaccine hesitancy [26]. We offered parents the opportunity to ask questions if there were any reservations about vaccinating their child. We assisted those who missed their vaccinations as well as re-establishing community demand through HPV “catch-up” campaigns. For those who preferred going to their healthcare provider to receive the vaccination in a traditional clinic setting, we coordinated the scheduling.

As our results show, middle schools are a feasible, effective setting for increasing HPV uptake. School settings are conducive to active adolescent engagement about HPV and HPV vaccination, promoting adolescent involvement in decision making, reducing needle-related fear and anxiety, and leading to more vaccination-literate adolescents [25,26]. Our successes with the pilot project allowed us to expand to five additional school districts. Our program increases access to the HPV vaccine and reaches a large, diverse population regardless of individual access to healthcare, and removes known barriers. For those exposed longer to the physician-led educational campaign (i.e., five additional school districts), baseline vaccination rates were higher. Extensive recovery efforts have been made to continue the progress of our HPV education and vaccination program throughout the coronavirus disease 2019 (COVID-19) pandemic. Our COVID-19 adaptations allowed for a safe environment for middle schoolers to get vaccinated. Although the HPV uptake increased throughout the COVID-19 pandemic, the rates did not increase as much as in the pilot project. Our results support how a grade-based vaccination strategy can lead to slightly higher uptake than an age-based strategy [21,22]. Since some of the students are transient, they can be older than their peers in the same grade. More studies are needed to explore the methods for standardizing estimates of HPV vaccine coverage so that programs can be appropriately evaluated.

This study had its limitations. First, information on baseline characteristics of students and parents, such as students’ race and ethnicity, socioeconomic status (SES), insurance status, parents’ education, country of birth, or knowledge and confidence in the HPV vaccines, was not collected. Additional comparisons evaluating these important characteristics could not be undertaken. Second, we did not have complete information on other vaccines or confirmation that other providers bundled HPV vaccines with other recommended vaccines. This could be an important future extension of our study. Next, the study population is transient, with some students changing schools during the study period. For simplification, we followed our baseline cohort at each middle school. Last, we are unable to account for all HPV vaccines. Although the vendor and schools shared updated information, it may not capture all vaccines received outside the school setting when parents fail to report vaccinations to the school. Future studies should explore issues, such as inadequate school-based health centers and vaccine billing as barriers to school-based HPV programs. Last, the study may have limited generalizability to Texas and the US. The school districts were not randomized, with implementation occurring in schools with the highest enrollment. There is also no national mandate for HPV vaccination.

## 5. Conclusions

School-based vaccination programs play an important role in increasing HPV vaccine uptake by reaching underserved adolescent populations who are most at risk for HPV-associated diseases. Through partnership with the RGV community and healthcare providers, our voluntary school-based vaccination program educated the community (parents, local providers, school board members, and staff) about the importance of HPV vaccines, removed access and transportation barriers, developed care coordination between local physicians and the RGV community, bundled HPV vaccines with other required vaccines when provided by our program, and increased HPV-UTD to 58.8%. Recommending HPV vaccine initiation at younger ages increases completion of the HPV vaccine series and providing access to HPV vaccines encourages on-time vaccination and completion. Increasing HPV vaccine uptake has the potential to decrease HPV-associated diseases in the area in the future.

## Figures and Tables

**Table 1:** Summary of HPV vaccine activities between August 2016 and August 31, 2022.

Variable ^1^	Total	RGCCISD Pilot Program	Expansion into PSJA ISD	Expansion into Roma ISD, Zapata ISD, San Isidro ISD
Number of middle schools	16	3	8	5
Number of HPV vaccine initiations delivered at school campus interventions	1549	497	677	375
Number of HPV vaccine completions delivered at school campus interventions	1042	578	378	86
Number of HPV vaccine initiations delivered by collaborating healthcare practices	18,172	4231	10,713	3228
Number of HPV vaccine completions delivered by collaborating healthcare practices	17,075	3936	10,829	2310
Number of school campus interventions	178	41	110	27

Note: HPV, human papillomavirus; PSJA ISD, Pharr-San Juan-Alamo Independent School District; Rio Grande City Consolidated Independent School District. ^1^ The counts represent the number of HPV vaccines provided and not the number of unique students who received HPV vaccine doses.

**Table 2:** Summary of demographic characteristics of the study population by gender were vaccinated directly or indirectly through our program (*n* = 19,951).

Variable	All Unique Students Vaccinated Directly or Indirectly ^1^ (*n* = 19,951)			All Unique Students Vaccinated Directly (*n* = 2145)		
	All (*n* = 19,951)	Females (*n* = 10,289)	Males (*n* = 9662)	All (*n* = 2145)	Females (*n* = 1074)	Males (*n* = 1071)
	*n* (%)	*n* (%)	*n* (%)	*n* (%)	*n* (%)	*n* (%)
Age Groups at Initiation						
9–10	4680 (23.5%)	2618 (25.4%)	2062 (21.3%)	222 (10.3%)	115 (10.7%)	107 (10.0%)
11–12	13,135 (65.8%)	6687 (65.0%)	6448 (66.7%)	1491 (69.5%)	750 (69.8%)	741 (69.2%)
13–14	1460 (7.3%)	673 (6.5%)	787 (8.1%)	319 (14.9%)	148 (13.8%)	171 (16.0%)
15–16	507 (2.5%)	239 (2.3%)	268 (2.8%)	64 (3.0%)	36 (3.4%)	28 (2.6%)
17+	169 (0.8%)	72 (0.7%)	97 (1.0%)	49 (2.3%)	25 (2.3%)	24 (2.2%)
Age at HPV Initiation						
Mean (SD)	11 (1.4)	11 (1.4)	11 (1.5)	12 (1.5)	12 (1.6)	12 (1.5)
Median (min, max)	11 (9, 21)	11 (9, 20)	11 (9, 21)	12 (9, 20)	11 (9, 20)	12 (9, 19)
School District						
RGCCISD	5583 (28.0%)	2916 (28.3%)	2667 (27.6%)	968 (45.1%)	471 (43.9%)	497 (46.4%)
PSJA ISD	11,390 (57.1%)	5905 (57.4%)	5485 (56.8%)	957 (44.6%)	498 (46.4%)	459 (42.9%)
Roma ISD	1523 (7.6%)	782 (7.6%)	741 (7.7%)	157 (7.3%)	73 (6.8%)	84 (7.8%)
Zapata ISD	1011 (5.1%)	481 (4.7%)	530 (5.5%)	58 (2.7%)	29 (2.7%)	29 (2.7%)
San Isidro ISD	103 (0.5%)	51 (0.5%)	52 (0.5%)	5 (0.2%)	3 (0.3%)	2 (0.2%)
Jim Hogg ISD	341 (1.7%)	154 (1.5%)	187 (1.9%)	0 (0.0%)	0 (0.0%)	0 (0.0%)
School District						
Elementary	6816 (342%)	3644 (35.4%)	3172 (32.8%)	253 (1.8%)	128 (11.9%)	125 (11.7%)
Middle school	12,079 (60.5%)	6147(59.7%)	5932 (61.4%)	1683 (78.5%)	838 (78.0%)	845 (78.9%)
High school	1056 (5.3%)	498 (4.8%)	558 (5.8%)	209 (9.7%)	108 (10.1%)	101 (9.4%)
Number of Doses						
1	8154 (40.9%)	3998 (38.9%)	4156 (43.0%)	1065 (49.7%)	512 (47.7%)	553 (51.6%)
2	9595 (48.1%)	5082 (49.4%)	4513 (46.7%)	911 (42.5%)	464 (43.2%)	447 (41.7%)
3+	2202 (11.0%)	1209 (11.8%)	993 (10.3%)	169 (7.9%)	98 (9.1%)	71 (6.6%)
Received the Initial HPV Dose from Our Program						
No	18,433 (92.4%)	9551 (92.8%)	8882 (91.9%)	627 (29.2%)	336 (31.3%)	291 (27.2%)
Yes	1518 (7.6%)	738 (7.2%)	780 (8.1%)	1518 (70.8%)	738 (68.7%)	780 (72.8%)
Received Other Vaccinations Bundled with HPV Vaccine ^2^						
No	Not available	Not available	Not available	617 (29.2%)	336 (31.3%)	291 (27.2%)
Yes	Not available	Not available	Not available	1528 (70.8%)	738 (68.7%)	780 (72.8%)
HPV-UTD ^3^						
No	8220 (41.2%)	4031 (39.2%)	4189 (43.4%)	1074 (50.1%)	519 (48.3%)	555 (51.8%)
Yes	11,731 (58.8%)	6258 (60.8%)	5473 (56.6%)	1071 (49.9%)	555 (51.7%)	516 (48.2%)
Age at HPV-UTD ^2^						
Mean (SD)	12 (1.6)	12 (1.6)	12 (1.6)	13 (1.4)	13 (1.5)	13 (1.4)
Median (min, max)	12 (9, 20)	11 (9, 19)	12 (9, 20)	12 (9, 19)	12 (9, 19)	13 (9, 18)
Days Between HPV Initiation and UTD						
Mean (SD)	403 (325.6)	404 (330.0)	401 (320.4)	480 (405.1)	478 (410.1)	482 (400.2)
Median (min, max)	291 (146, 2968)	286 (146, 2967)	300 (146, 2968)	324 (146, 2855)	310 (146, 2855)	339 (150, 2843)

Note: HPV, human papillomavirus; ISD, independent school district, Max, maximum; Min, minimum; SD, standard deviation. ^1.^ This includes both students vaccinated within our program and those vaccinated outside the program because of HPV awareness and community provider collaboration. ^2.^ For students vaccinated outside, we do not know if they received other vaccinations bundled with the HPV vaccine.^3.^ HPV up-to-date (HPV-UTD) was defined in accordance with the 2016 ACIP guidelines as either (1) receipt of 3 or more doses or (2) receipt of 2 doses of the HPV vaccine, with the first shot administered before age 15 years, and the time between the first and second dose was at least 5 months minus 4 days.

**Table 3:** Multivariate analysis of factors associated with HPV-UTD among all students vaccinated (indirectly and directly: *n* = 19,951) and students vaccinated directly through our school-based program (*n* = 2145).

Variable		All Students Vaccinated Indirectly and Directly ^1^ (*n* = 19,951)		Students Vaccinated Directly (*n* = 2145)	
		OR (95% CI)	*p*-Value	OR (95% CI)	*p*-Value
Age at initiation (1-year increase)		0.676 (0.641–0.712)	<0.0001	0.626 (0.549–0.714)	<0.0001
Gender	Female	1.000		1.000	
	Male	0.902 (0.833–0.976)	0.0103	0.792 (0.642–0.976)	0.0288
School grade at initiation	Elementary	1.385 (1.219–1.573)	<0.0001	1.008 (0.676–1.505)	0.9671
	Middle school	1.000		1.000	
	High school	1.96 (1.097–1.776)	0.0068	0.517 (0.285–0.937)	0..0296
School district	RGCCISD	1.000		1.000	
	PSJA ISD	1.205 (1.091–1.331)	0.0002	1.141 (0.861–1.512)	0.3589
	Roma ISD	0.623 (0.521–0.743)	<0.0001	1.679 (1.030–2.735)	0.0375
	San Isidro ISD	1.445 (0.833–2.507)	0.1904	0.929 (0.033–26.408)	0.9658
	Zapata ISD	1.701 (1.422–2.034)	<0.0001	1.094 (0.373–3.212)	0.8702
	Jim Hogg ISD	0.976 (0.741–1.285)	0.8621		
Received the initial HPV dose through our program	Yes	1.000			
	No	0.636 (0.544–0.743)	<0.0001		
Intervention year ^2^	2016	1.000			
	2017	0.694 (0.595–0.810)	<0.0001	1.000	
	2018	0.515 (0.442–0.601)	<0.0001	0.418 (0.199–0.882)	0.022
	2019	0.226 (0.194–0.264)	<0.0001	0.226 (0.110–0.465)	<0.0001
	2020	0.142 (0.118–0.171)	<0.0001	0.089 (0.041–0.193)	<0.0001
	2021	0.046 (0.035–0.060)	<0.0001	0.050 (0.024–0.106)	<0.0001
	2022	0.007 (0.003–0.013)	<0.0001	0.008 (0.003–0.017)	<0.0001

^1.^ This includes both students vaccinated within our program and those vaccinated outside the program because of increased HPV awareness and collaborations with community healthcare providers. ^2.^ For students vaccinated indirectly or directly, intervention year is the first dose received after 31 August2016 (start of our first program in RGV). This could be the initiation dose or any follow-up dose. For students who were vaccinated directly, intervention year is the year that the first dose was received from our school-based program (could be the initiation dose or any follow-up dose).

## Data Availability

The data that was used and/or analyzed are available upon request from the corresponding author.

## Abbreviations

- Advisory Committee on Immunization Practices (ACIP)
- CDC: Centers for Disease Control and Prevention
- COVID-19: coronavirus Disease 2019
- HPV: human Papillomavirus
- ISD: independent school district
- PSJA ISD: Pharr-San Juan-Alamo Independent School District
- RGV: Rio Grande Valley
- RGCCISD: Rio Grande City Consolidated Independent School District
- SD: standard deviation
- TDAP: tetanus, diphtheria (TD), or tetanus, diphtheria, and pertussis
- US: United States
- UTD: up-to-date
- VFC: vaccines for children

## Appendix A

The appendix contains detailed information on HPV vaccination data. Table A1 provides details on the students from each school district who received the HPV vaccinations directly through our school-based program.

**Table A1:** Descriptive of the students by school district who received HPV vaccines directly through our school-based program (*n* = 2145).

	All			RGCCISD			PSJA ISD			Roma ISD			Zapata ISD			San Isidro ISD		
	Unique Students Reached (as of 31 August 2022) *n* = 2145			Unique Students Reached (as of 31 August 2022) *n* = 968			Unique Students Reached (as of 31 August 2022) *n* = 957			Unique Students Reached (as of 31 August 2022) *n* = 157			Unique Students Reached (as of 31 August 2022) *n* = 58			Unique Students Reached (as of 31 August 2022) *n* = 5		
	All	Females *n* = 1074	Males *n* = 1071	All	Females *n* = 471	Males *n* = 497	All	Females *n* = 498	Males *n* = 459	All	Females *n* = 73	Males *n* = 84	All	Females *n* = 29	Males *n* = 29	All	Females *n* = 3	Males *n* = 2
Age groups at Initiation																		
9–10	222 (10.3%)	115 (10.7%)	107 (10.0%)	93 (9.6%)	45 (9.6%)	48 (9.7%)	124 (13.0%)	68 (13.7%)	56 (12.2%)	3 (1.9%)	0	3 (3.6%)	1 (1.7%)	1 (3.4%)	0	1 (20.0%)	1 (33.3%)	0
11–12	1491 (69.5%)	750 (69.8%)	741 (69.2%)	731 (75.5%)	366 (77.7%)	365 (73.4%)	600 (62.7%)	306 (61.4%)	294 (64.1%)	113 (72.0%)	56 (76.7%)	57 (67.9%)	45 (77.6%)	20 (69.0%)	25 (86.2%)	2 (40.0%)	2 (66.7%)	0
13–14	319 (14.9%)	148 (13.8%)	171 (16.0%)	121 (12.5%)	49 (10.4%)	72 (14.5%)	159 (16.6%)	80 (16.1%)	79 (17.2%)	25 (15.9%)	11 (15.1%)	14 (16.7%)	12 (20.7%)	8 (27.6%)	4 (13.8%)	2 (40.0%)	0	2 (100.0%)
15–16	64 (3.0%)	36 (3.4%)	28 (2.6%)	15 (1.5%)	7 (1.5%)	8 (1.6%)	41 (4.3%)	24 (4.8%)	17 (3.7%)	8 (5.1%)	5 (6.8%)	3 (3.6%)	0	0	0	0	0	0
17+	49 (2.3%)	25 (2.3%)	24 (2.2%)	8 (0.8%)	4 (0.8%)	4 (0.8%)	33 (3.4%)	20 (4.0%)	13 (2.8%)	8 (5.1%)	1 (1.4%)	7 (8.3%)	0	0	0	0	0	0
Age at HPV Initiation																		
Mean (SD)	12 (1.5)	12 (1.6)	12 (1.5)	12 (1.2)	12 (1.2)	12 (1.3)	12 (1.8)	12 (1.9)	12 (1.6)	12 (1.8)	12 (1.5)	12 (2.0)	12 (1.0)	12 (1.2)	12 (0.8)	12 (1.6)	11 (0.6)	14 (0.7)
Median (min, max)	12 (9, 20)	11 (9, 20)	12 (9, 19)	11 (9, 19)	11 (9, 18)	12 (9, 19)	12 (9, 20)	11 (9, 20)	12 (9, 18)	12 (9, 20)	12 (11, 20)	12 (9, 18)	12 (9, 14)	12 (9, 14)	12 (11, 14)	11 (10, 14)	11 (10, 11)	14 (13, 14)
School Grade at Initiation																		
Elementary school	253 (11.8%)	128 (11.9%)	125 (11.7%)	109 (11.3%)	54 (11.5%)	55 (11.1%)	111 (11.6%)	61 (12.2%)	50 (10.9%)	31 (19.7%)	11 (15.1%)	20 (23.8%)	1 (1.7%)	1 (3.4%)	0	1 (20.0%)	1 (33.3%)	0
Middle school	1683 (78.5%)	838 (78.0%)	845 (78.9%)	812 (83.9%)	391 (83.0%)	421 (84.7%)	712 (74.4%)	365 (73.3%)	347 (75.6%)	105 (66.9%)	56 (76.7%)	49 (58.3%)	51 (87.9%)	24 (82.8%)	27 (93.1%)	3 (60.0%)	2 (66.7%)	1 (50.0%)
High school	209 (9.7%)	108 (10.1%)	101 (9.4%)	47 (4.9%)	26 (5.5%)	21 (4.2%)	134 (14.0%)	72 (14.5%)	62 (13.5%)	21 (13.4%)	6 (8.2%)	15 (17.9%)	6 (10.3%)	4 (13.8%)	2 (6.9%)	1 (20.0%)	0	1 (50.0%)
Number of HPV Vaccine Doses																		
1	1065 (49.7%)	512 (47.7%)	553 (51.6%)	352 (36.4%)	158 (33.5%)	194 (39.0%)	542 (56.6%)	275 (55.2%)	267 (58.2%)	112 (71.3%)	50 (68.5%)	62 (73.8%)	54 (93.1%)	26 (89.7%)	28 (96.6%)	5 (100.0%)	3 (100.0%)	2 (100.0%)
2	911 (42.5%)	464 (43.2%)	447 (41.7%)	524 (54.1%)	263 (55.8%)	261 (52.5%)	344 (35.9%)	179 (35.9%)	165 (35.9%)	41 (26.1%)	21 (28.8%)	20 (23.8%)	2 (3.4%)	1 (3.4%)	1 (3.4%)	0	0	0
3+	169 (7.9%)	98 (9.1%)	71 (6.6%)	92 (9.5%)	50 (10.6%)	42 (8.5%)	71 (7.4%)	44 (8.8%)	27 (5.9%)	4 (2.5%)	2 (2.7%)	2 (2.4%)	2 (3.4%)	2 (6.9%)	0	0	0	0
Received the initial HPV dose from our program																		
No	627 (29.2%)	336 (31.3%)	291 (27.2%)	304 (31.4%)	160 (34.0%)	144 (29.0%)	280 (29.3%)	153 (30.7%)	127 (27.7%)	39 (24.8%)	20 (27.4%)	19 (22.6%)	4 (6.9%)	3 (10.3%)	1 (3.4%)	0	0	0
Yes	1518 (70.8%)	738 (68.7%)	780 (72.8%)	664 (68.6%)	311 (66.0%)	353 (71.0%)	677 (70.7%)	345 (69.3%)	332 (72.3%)	118 (75.2%)	53 (72.6%)	65 (77.4%)	54 (93.1%)	26 (89.7%)	28 (96.6%)	5 (100.0%)	3 (100.0%)	2 (100.0%)
Received Other Vaccinations Bundled with the HPV Vaccine																		
No	617 (28.8%)	307 (28.6%)	310 (28.9%)	380 (39.3%)	191 (40.6%)	189 (38.0%)	143 (14.9%)	67 (13.5%)	76 (16.6%)	45 (28.7%)	23 (31.5%)	22 (26.2%)	48 (82.8%)	25 (86.2%)	23 (79.3%)	1 (20.0%)	1 (33.3%)	0
Yes	1528 (71.2%)	767 (71.4%)	761 (71.1%)	588 (60.7%)	280 (59.4%)	308 (62.0%)	814 (85.1%)	431 (86.5%)	383 (83.4%)	112 (71.3%)	50 (68.5%)	62 (73.8%)	10 (17.2%)	4 (13.8%)	6 (20.7%)	4 (80.0%)	2 (66.7%)	2 (100.0%)
HPV-UTD																		
No	1074 (50.1%)	519 (48.3%)	555 (51.8%)	355 (36.7%)	161 (34.2%)	194 (39.0%)	548 (57.3%)	279 (56.0%)	269 (58.6%)	112 (71.3%)	50 (68.5%)	62 (73.8%)	54 (93.1%)	26 (89.7%)	28 (96.6%)	5 (100.0%)	3 (100.0%)	2 (100.0%)
Yes	1071 (49.9%)	555 (51.7%)	516 (48.2%)	613 (63.3%)	310 (65.8%)	303 (61.0%)	409 (42.7%)	219 (44.0%)	190 (41.4%)	45 (28.7%)	23 (31.5%)	22 (26.2%)	4 (6.9%)	3 (10.3%)	1 (3.4%)	0	0	0
Age at HPV-UTD																		
Mean (SD)	13 (1.4)	13 (1.5)	13 (1.4)	12 (1.2)	12 (1.2)	12 (1.2)	13 (1.7)	13 (1.7)	13 (1.6)	13 (1.2)	13 (0.7)	14 (1.5)	13 (1.7)	12 (2.1)	13 (.)	*n*/A	*n*/A	*n*/A
Median (min, max)	12 (9, 19)	12 (9, 19)	13 (9, 18)	12 (9, 18)	12 (9, 18)	12 (9, 18)	12 (9, 19)	12 (9, 19)	13 (9, 18)	13 (11, 18)	13 (12, 15)	13 (11, 18)	13 (10, 14)	13 (10, 14)	13 (13, 13)			
Days between Initiation and UTD																		
Mean (SD)	480 (405.1)	478 (410.1)	482 (400.2)	393 (297.0)	395 (314.1)	392 (279.1)	564 (487.3)	563 (495.9)	567 (478.4)	889 (460.5)	809 (345.7)	972 (552.3)	525 (371.0)	390 (310.1)	932 (.)	*n*/A	*n*/A	*n*/A
Median (min, max)	324 (146, 2855)	310 (146, 2855)	339 (150, 2843)	268 (149, 2341)	260 (149, 2016)	272 (150, 2341)	374 (146, 2855)	364 (146, 2855)	403 (167, 2843)	874 (160, 2523)	829 (160, 1469)	875 (188, 2523)	489 (191, 932)	231 (191, 747)	932 (932, 932)			

Note: HPV, human papillomavirus; ISD, independent school district, Max, maximum; Min, minimum; SD, standard deviation.
